# High-Value Utilization of Marine Biological Resources

**DOI:** 10.3390/foods12224054

**Published:** 2023-11-07

**Authors:** Rongfeng Li, Pengcheng Li

**Affiliations:** 1 CAS and Shandong Province Key Laboratory of Experimental Marine Biology, Center for Ocean Mega-Science, Institute of Oceanology, Chinese Academy of Sciences, No. 7 Nanhai Road, Qingdao 266071, China; pcli@qdio.ac.cn; 2 Laboratory for Marine Biology and Biotechnology, Qingdao National Laboratory for Marine Science and Technology, Qingdao 266237, China

## 1. Introduction

The ocean covers 71% of the surface of our planet and comprises a diverse variety of biological resources—a combination of marine animals, marine plants, and marine microorganisms that have economic value for human beings. Some of them can be used directly as food, and others may be developed into high-value healthy food, marine drugs, or novel materials.

The world’s population reached 8 billion people and will reach 9.7 billion in 2050 [1] (https://www.un.org/en/global-issues/population). The global food supply will face a significant challenge. Seafood plays an important role in the global food supply system. In 2020, global production of marine animals and marine algae was estimated at 112 million tonnes and ~34 million tonnes (wet weight), respectively, as reported by the Food and Agriculture Organization of the United Nations (FAO) [2]. Seafood provides high-quality proteins and other nutrients for human beings. However, the edible portions of seafood are usually very low. Fish or Crustacea marine animals processing by-products or waste is even up to 70%. Therefore, the valorization of seafood by-products can also increase the global food supply [3].

Currently, an increasing number of people are concerned about the nutritional and healthy benefits of food, rather than only being a source of energy. Marine biological resources contain a large number of bioactive molecules that are beneficial to human health. The development and application of novel technologies for extracting nutrients and bioactive ingredients from marine biological resources will produce a diversity of high-value nutritional food, healthy food, special medical food, cosmetic products, medical materials, and marine drugs. Therefore, high-value utilization of marine biological resources can also benefit human health and the global blue economy.

## 2. High-Value Utilization of Marine Biological Resources

Marine biological resources include various seafood, seafood processing by-products, and inedible marine microbial resources. Seafood, such as marine fish, marine shrimp, marine crab, scallops, oysters, squid, jellyfish, sea cucumber, sea urchin, kelp, wakame, sargassum, and seaweeds, etc., contains several proteins, enzymes, peptides, polysaccharides, omega-3 long-chain polyunsaturated fatty acids, astaxanthin, taurine, saponin, vitamins, etc. [4]. However, the value of seafood is not fully realized if it is only directly consumed as food. Therefore, deep-processing of seafood, valorizing seafood processing by-products and low-value seafood, and developing and applying marine microbial resources will be high-value uses of marine biological resources.

Although seafood contains many nutrients and bioactive substances, it may not be completely absorbed and fully display its functions in our body without further processing. The deep-processing of seafood is an application of advanced processing technologies, including supercritical fluid extraction technology, microencapsulation technology, ultra-micro pulverization technology, membrane separation technology, enzyme technology, fermentation technology, etc., for the production of a diversity of high-value healthy food, functional food, and special medical food.

Seafood processing by-products are usually discarded as garbage or made into low-value animal feed or fertilizer, which may also cause environmental pollution and marine biological resource waste. Seafood processing by-products still contain many bioactive components, such as residual proteins, collagen in fish skin, chitin in the shells of shrimp and crab, chondroitin sulfate in fish cartilage, enzymes in animal visceral organs, fucoidan in algae waste, minerals in the shells of scallops and oysters, etc. [4]. These active components can also be extracted from those seafood processing by-products by using modern food processing technologies to produce high-value marine healthy food, marine drugs, and functional materials. Moreover, there are many low-value species of marine animals and plants that people do not prefer to eat as a result of their small size or poor taste, such as fish *Engraulis ringens*, shrimp *Antarctic krill*, seaweed *Ulva prolifera*, and jellyfish. However, those marine animals and plants are also rich in plenty of bioactive molecules, which can be used as raw materials to produce high-value products such as fish peptides, surimi, shrimp oil with omega-3, EPA, and DHA, chitin/chitosan/chitooligosaccharide, jellyfish peptides, etc. [5].

Although marine microorganisms cannot be directly used as food for humans, they contain many kinds of special enzymes as a result of the harsh living conditions with high salinity, extensive pH, a wide temperature range, and high pressure. Marine-derived enzymes such as chitinase, chitosanase, alginate lyase, agarase, carrageenase, α-amylase, xylanase, lipases, proteases, and collagenases can specifically recognize and degrade substrates into low-molecular-weight and highly active oligosaccharides, monosaccharides, peptides, or lipids, which can be applied to food, healthcare, cosmetics, agriculture, and the pharmaceutical industry [6,7].

At present, our understanding, development, and utilization of marine biological resources are still very limited and face many challenges. We believe that with the progress of scientific research and the development of new biotechnologies, the high-value utilization of marine biological resources will further benefit human health and life in the future.

This Special Issue aims to publish high-quality articles on the high-value utilization of marine biological resources from a wide range of aspects, including deep-processing of seafood, high-value utilization of seafood processing by-products and low-value seafood, and the identification and characterization of enzymes from marine organisms for potential application in the food industry to produce food materials or additives such as chitosan, carrageenan, and gelatin.

## References

[1] United Nations, Department of Economic and Social Affairs, Population Division (2022). World Population Prospects 2022: Ten Key Messages.

[2] FAO (2022). The State of World Fisheries and Aquaculture 2022.

[3] Sandström V., Chrysafi A., Lamminen M., Troell M., Jalava M., Piipponen J., Siebert S., van Hal O., Virkki V., Kummu M. (2022). Food system by-products upcycled in livestock and aquaculture feeds can increase global food supply. Nat. Food.

[4] Ozogul F., Cagalj M., Šimat V., Ozogul Y., Tkaczewska J., Hassoun A., Kaddour A.A., Kuley E., Rathod N.B., Phadke G.G. (2021). Recent developments in valorisation of bioactive ingredients in discard/seafood processing by-products. Trends Food Sci. Technol..

[5] Li P. (2020). Research progress in high-value utilization of marine biological resources. Oceanol. Limnol. Sin..

[6] Sun H., Gao L., Xue C., Mao X. (2020). Marine-polysaccharide degrading enzymes: Status and prospects. Compr. Rev. Food Sci. Food Saf..

[7] Ghattavi S., Homaei A. (2023). Marine enzymes: Classification and application in various industries. Int. J. Biol. Macromol..

